# Relationships Between Medical Doctors’ Personality Traits and Their Professional Risk Perception

**DOI:** 10.3390/bs10010006

**Published:** 2019-12-19

**Authors:** Nataliya Bogacheva, Tatiana Kornilova, Elizaveta Pavlova

**Affiliations:** 1 Clinical Psychology Department, Sechenov First Moscow State Medical University (Sechenov University), 8 Trubetskaya Street, Bldg. 2, Moscow 119991, Russia; 2 General Psychology Department, Lomonosov Moscow State University, Mokhovaya Street, 11/9, Moscow 125009, Russia; tvkornilova@mail.ru; 3 Educational Psychology and Pedagogy Department, Lomonosov Moscow State University, Mokhovaya Street, 11/9, Moscow 125009, Russia; pavlova.lisa@gmail.com

**Keywords:** medical risks, medical decision-making, personality traits, Big Five, tolerance for uncertainty, cognitive representations of risk

## Abstract

Medical decision-making is often related to risk and uncertainty, but existing research does not offer a comprehensive approach to this matter. We discuss the necessity to study cognitive representations of risks (CRRs), which we define as the subject’s images of risky situations, possible outcomes, and alternative decisions. The psychometric approach towards risk assessment often involves the evaluation of different risks, but we aim to create such a list from medical professionals’ expert knowledge. Via qualitative analysis, CRRs were obtained from interviews with practicing doctors from Russia (N = 24). The list includes 21 risks from real-life medical practice, with seven aspects for numerical evaluation each. Then, practicing doctors (N = 64) evaluated CRRs along with filling risk-related personality traits questionnaires: Personal Decision-Making Factors Questionnaire, Melbourne Decision Making Questionnaire, Ten Item Personality Measure, and Budner’s Intolerance of Ambiguity Scale. A correlational analysis showed interconnections between most CRRs aspects, with predictability and negative outcome probability seemingly being the central aspects of the risk assessment. CRRs aspects were also found to be gender- and experience-specific, with female doctors and younger specialists being more sensitive to professional risks. Personality traits in relation to CRRs aspects, medical experience and gender are also discussed.

## 1. Introduction

Medical professionals face uncertainty and risks in their work on an everyday basis. Decision-making under the circumstances of limited time and a lack of necessary information is always risky in a subjective way. In medicine, it can also lead to the risk of harming the patient. Several recent studies have suggested that while there is a notion that uncertainty and tolerance for uncertainty play a crucial role in medical decision-making, a comprehensive approach towards understanding and assessing uncertainty in healthcare has yet to be framed [1,2].

Many aspects of medical decision-making, as well as medical risk assessment, have a rich history of psychological research, mostly within cognitive and organizational psychology frameworks. A few studies have addressed doctors’ susceptibility towards the “framing-effect” (in terms of Kahneman’s and Tversky’s “prospect theory”) [3,4], as well as other cognitive biases and heuristics that are usually seen as detrimental but sometimes are seen as beneficial for actual decision-making [5]. Clinical decisions have often been found to be based on induction, as opposed to the more rational hypothetico-deductive methods [6], with medical professionals relying more on their experience-based judgments than risk assessment scales that are designed to make those decisions more objective [7,8]. Doctors’, nurses’ and medical students’ personality traits that are related to decision-making and risk-taking have also been the subject of psychological research [9,10,11,12], and it has been shown that surgeons, in addition to being more intolerant to uncertainty [9], are more prone to risk-taking in a gambling task, based on the game theory [11], which indicates a possible difference between self-perception of personality and actual behavior. Lawton et al. argued that doctors’ tolerance for uncertainty, as well as their professional experience, can be beneficial for their patients’ safety [13]. Motivation has also been shown to be related to the choice preferences in medical risk-related verbal tasks [14]. However, attitudes towards uncertainty and risk-readiness (as the ability to decide under uncertainty) should be considered as the most probable personality-based regulators of the decision-making.

We suggest that a productive avenue to overcome and bridge the existing gaps and discrepancies in the field’s understanding of medical decision-making under uncertainty and risk lies within building a comprehensive approach that can reconcile different research paradigms and emphasize the role of the thinking subject’s intellectual activity. Such an approach is possible within the concept of the unified functioning of a person’s intellectual and personal potential and the dynamic multi-level regulation of human activity under uncertainty [15,16]. This approach includes identifying the relationships between medical workers’ personality traits and the specifics of their perception of and relation towards their professional risks. 

The research of risk perception in psychology has led to the understanding of cognitive representations of risks and as subjective, complex, and mostly conscious [17,18]. Heller et al. argued that residents at different levels of hospital training are not only prone to making heuristic-based decisions but also are more likely to use heuristics in medical tasks comparing to non-medical ones [19]. Thus, medical risks that are perceived by medical professionals should be distinguished and researched separately from the risks that are unrelated to their professional activities.

While the general idea of cognitive representations of risks as the subject’s image of a risky situation, its possible outcomes and alternative decisions emerged from the works of Kahneman and Tversky [3], and this idea has yet to be integrated into the analysis of a comprehensive system of the personality-based regulation of the decision-making process [16]. There is also a need to establish which personality traits play a distinct role in the decision-making regulation and which are more distant from decision processes under uncertainty. Personality traits can also be either relatively stable, like the Big Five personality traits, or show dynamic properties concerning uncertainty, like tolerance or intolerance for ambiguity, risk-readiness, or uncertainty coping mechanisms.

However, the lack of scales assessing the perception of medical risks does not allow us to straightforwardly set the task of identifying the relationship between personality and cognitive representations of risk. To fill this gap, we propose a medical risk assessment procedure based on a scoring system. At the same time, we also identify risky situations that medical doctors themselves consider significant from the point of their professional activities.

The psychometric approach towards risk perception research is based on the assessment of specific types of risk by particular people, e.g., experts or novices in different fields. In those types of studies, lists of various risks and different respondents’ report forms are used. For example, medical risks can be estimated in the numbers of deaths per disease point, in the probabilistic assessment of the event occurrence, etc. [20]. Gigerenzer discussed, in detail, the risks of the wrong diagnosis due to the specific form of medical data (either probabilistic or natural frequencies-based) [18]. However, in most of those studies, the participants were provided the list of risks. On the other hand, we wanted to obtain the types of medical risks for future assessment from the doctors themselves via interviews. Thus, the aims of the study were (1) to identify personality specifics of medical doctors via psychodiagnostic methods, (2) to create cognitive representations of medical risks scale with risks being present the same way as in real-life medical practice, and (3) to link those cognitive representations of medical risk with doctors’ personality traits. 

The study, therefore, tested the following hypotheses:

**Hypotheses 1.**  *Attitudes towards ambiguity, uncertainty coping strategies, risk-readiness and the Big Five personality traits are related to different aspects of the cognitive representation of risks, obtained through the interviews with medical professionals.*

**Hypotheses 2.**  *Cognitive representations of risk are different for medical practitioners with different professional experiences and of a different gender.*

## 2. Materials and Method

### 2.1. First Stage of the Research: Identifying Cognitive Representations of Risk (CRRs) in Medicine

#### 2.1.1. Participants and Procedure

Practicing doctors from Russia voluntarily took part in the interviews on medical risk perception. The interviews were taken during individual face-to-face encounters and consisted of two parts. The doctors were asked to describe a real-life, professionally significant, risky situation(s) that happened to them or their colleagues, following Flanagan’s critical incident technique [21]. The participants were asked to describe the situation(s), explain why they considered them to be risky, describe their behavior and other considered options, describe their reasoning and then evaluate the effectiveness of their choice and share the outcomes. According to Sternberg [22], this method is useful for building rating scales and situational judgment tests for professionals, matching our goals. A semi-structured interview followed that consisted of professional risk related questions, such as “Can you describe your job as a risky one?” and “What risks can doctors face in their practice?” The interviews were recorded by writing and voice-recording.

The recruitment process involved “snowball sampling” among the doctors in two different medical facilities in Moscow. The de facto the sample included professionals with various backgrounds and professional experiences; their definitions as surgeons and ophthalmologists only represented their current status (e.g., among surgeons, there was a former military doctor, a former emergency doctor, etc.). With 20 participants (10 from each facility), we reached theoretical saturation for the question-based part of the interview, as the answers turned to be repetitive. Critical incidents were still different story-wise but involved the same risk types as the interviews, according to primary qualitative analysis. Following the common practice [23], we added four more participants to ensure saturation. They were recruited separately from different medical facilities and were interviewed by a different interviewer. Those four included a younger doctor with one year of medical practice, a therapist from an outpatient hospital, a neurosurgeon, and a surgeon–oncologist. We supposed that diverse participants could bring new data, but they did not, so we agreed on data saturation. 

Overall, 24 medical doctors aged 25–68 (M = 41.33, SD = 11.17) who had been working for 1–39 years, with 17 males and 7 females, 13 surgeons, 10 ophthalmologists, and 1 therapist, participated (M = 16.21; SD = 10.0) in clinics in roles that required full-fledged medical decision making.

The study was conducted according to the principles of the Declaration of Helsinki and was approved by the local ethics committee. The participants provided their written informed consent for data gathering, processing and publication in an anonymous generalized form and for scientific purposes.

#### 2.1.2. Methods

The interview protocols were qualitatively analyzed by two psychologists with the meaning condensation method [24]. This method involves the transformation of the raw text, transformed by the participants into shorter and simpler messages, retaining the same main ideas and topics. The experts performed the meaning condensation separately and then combined the results while supervised by the third expert to ensure consistent conclusions. The identification and categorization of risk-related units both from the incidents and the structured interviews were also made within grounded theory methodology (open and axial coding procedures) [25]. Open coding involves the basic categorization of each meaningful text item (also related to the main topic of the research, e.g., medical risks). It is used to determine the range in which specific categories exist in the analyzed texts. Open coding also helps to develop questions for future interviews, if needed, so it was started early in the research. The axis coding process involves the choice of several of the most important (common and meaningful) categories from the previous step and sets them as centers of the coding with other categories being subordinate towards them. The axis was identified from the source material and not from some preexisting theory, and the relationships between them were established though the data processing by the experts. The decision about the axis and other coding results were also collegial.

### 2.2. Second Stage of the Research: Establishing Relationships Between Traits and CRRs

#### 2.2.1. Participants

Practicing doctors (N = 64) from Moscow, Russia, working in state and private clinics were recruited on a voluntary basis, with 27 males and 37 females aged 24–73 (M = 40.94; SD = 12.41) who had been practicing for 1–50 years (M = 15.76; SD = 11.77) and who had been specializing in ophthalmology (N = 27), general and vascular surgery (N = 16), therapy (N = 5), orthopedics (N = 4) and other specialists (N = 12). 

#### 2.2.2. Procedure

The participants successively and individually completed psychodiagnostic questionnaires on personality traits in pen-and-paper variants and then filled the specially designed risk assessment scale, established through the 1st stage of the study.

#### 2.2.3. Methods and Outcome Measures

- Scales for cognitive representations of risks (CRRs) evaluation (see Section 3.1).
- Personal Decision-Making Factors Questionnaire [15]: This questionnaire consists of 21 yes-or-no statements and measures two sub-scales: personal risk-readiness, the ability to make decisions in uncertain situations with a lack of necessary information, and rationality, personal preference to get as much information as possible to increase awareness before acting.
- Melbourne Decision Making Questionnaire [26], in the Russian adaptation [27]. The Russian variant retains the 22-statements structure and measures 4 coping patterns: vigilance, hypervigilance, buck-passing, and procrastination. Vigilance is the only productive coping mechanism, as it is associated with orientation and caution; others are unproductive. Buck-passing is avoiding independent decision-making, procrastination is an unjustified postponement of the decision-making, and hypervigilance is an excessive fluctuation between alternatives.
- Ten Item Personality Measure (TIPI) [28], in the Russian adaptation [29], measuring the Big Five personality traits: extraversion, emotional stability, conscientiousness, agreeableness, and openness.
- Budner’s Intolerance of Ambiguity Scale [30], in the Russian adaptation [31]. The Russian scale consists of 13 statements with a 7-point Likert scale. In has 2 sub-scales: tolerance for ambiguity, which is a tendency to perceive ambiguous and uncertain situations as desirable, and intolerance for ambiguity, which is the rejection of ambiguous and uncertain situations in the strive for clarity.

#### 2.2.4. Statistical Methods

All calculations were made in IBM SPSS ver. 22. Due to the sample size, non-parametric statistical methods were applied: Mann–Whitney U test, Kruskal–Wallis H test with post hoc Dunn test for pairwise comparisons, and Spearman’s rank correlation coefficient (rho). 

## 3. Results

### 3.1. Qualitative Research Results (results of the first stage)

Through the meaning condensation method, 422 primary text units related to medical risks were obtained. Those units were gathered around a higher level axis: risks (risky situations from medical practice, something that can happen)—125 units; risk sources (something leading to risks)—133 units; risk-reduction strategies—88 units; risk probability—41 units; risk predictability—35 units. For cognitive representations of risks scaling, we were interested in the first group, risks (see Table 1).

“Medicine as science related risks” and “abstract risk factors” (italicized in Table 1) were excluded from the list for being either too general for doctors to cope with (e.g., science imperfections or chance) or restricted to narrow practices like testing new procedures. On the contrary, we aimed to develop a list of risks that were potentially understandable for all medical professionals. The final variant included 21 risks that were the common examples from the interviews and were related to the secondary level subcategories. Those risks were randomized (see Appendix A) and followed by the instruction to assess (evaluate from 0 to 100) seven aspects of them: the perceived riskiness of the situation, its predictability, its probability in medical practice in general and in the respondent’s medical practice, its perceived emotion depth if it happens, and the probability of positive and negative outcomes in each situation. Thus, our participants could give a quantitative evaluation for the qualitative parameters same as in many works within the psychometric approach [20], but they could do so with risks based on the interviews with other practitioners. However, those scales were not intended to be the items of a standardized questionnaire.

### 3.2. Correlational Analysis

Most of the evaluated CRRs aspects showed significant, albeit imperfect, intercorrelations, thus making them related to one another without being redundant. While doctors did sometimes merge riskiness with risk predictability and probability, as seen during the interviews, correlations indicated that they were distinguishable if addressed directly (see Table 2).

CRRs measurements showed only a few correlations with personality inventories (see Table 3). 

Thus, positive outcomes estimation seemingly stands out from other CRRs, as it found to be was moderately related to negative outcome prediction. Negatively correlated with rationality and vigilance, positive outcome probability perception might be related to some “easy-going” attitudes and did not follow the pattern of other CRRs aspects. Perceived predictability and perceived negative outcome probability consolidated the greatest number of moderately strong correlations, seemingly being the core CRRs aspect along with emotional depth. This somewhat matches previous research on risk perception, where the probability of harm and expected mortality have been seen as the core risk-evaluation characteristics for experts, unlike those used by laypeople, who tend to have broader ideas of risks [32].

### 3.3. Personality Traits and CRRs Aspects Evaluation Scores in Relation to Professional Experience and Gender

Personality measurements showed no significant differences between male and female doctors, which slightly contradicts the previous findings [15,16,27,29] but could also be attributable to the modest statistical power of our study. However, it could also indicate that the professional selection or profession-related development of personality traits in medical practitioners in Russia exceeds their gender-related specifics. This, however, requires further investigation. On the other hand, CRRs evaluation scores were found to be gender-specific (see Table 4 for significant differences).

Female doctors evaluated most of CRRs aspects higher than the males, which matches previous findings in psychometric risk research, where women have been found to generally perceive risks higher than men [32].

Due to the professional trajectory of medical doctors in Russia, age and working experience are strongly correlated (in our sample, ρ = 0.975, *p* < 0.001), thus making it impossible to study them separately. We divided our sample into three tertile groups: young doctors (<35 years old, ≤9 years in practice), experienced doctors (aged 35–43; 10–17 years in practice), and older doctors (>43 years old, >17 years in practice), with a semi-equal gender ratio in each group (see Table 5 for differences).

In CRRs, perceived probabilities and emotional depth seem to be experience- and/or age-related—the older doctors gave lower values for those parameters. Younger doctors might be more sensitive towards risks (probably due to the lack of experience), while their older, experienced colleagues might have already encountered most of those risks. This result also follows known differences between experts and non-experts in risk-assessment [32]. Younger doctors are also less rational and more risk-ready than their older colleagues. Previously, we found doctors to be less risky and more rational than medical students [33]. Thus, an increase in rationality and a decrease in risk-readiness seems to be the profession-specific personality trait shift, a shift which could also be age-related. The higher openness of younger doctors is consistent with the Big Five traits’ age-related changes; however, a higher extraversion in older doctors in our sample seemed to be unpredicted and was possibly an artifact [34].

## 4. Discussion

Based on the results, we accept both our hypotheses. Firstly, relationships between personality traits and the subjective cognitive assessments of various risks (in the professional activity of a doctor) have been established. Secondly, differences in risk assessments by gender and medical experience have been identified. Thus, we believe that the set goals have been achieved and medical risk perception received the required attention due to its subjective importance.

Currently, the psychological understanding of risk is moving away from seeing it as a unitary construct that linked with impulsivity and lack of control [35]. Indeed, it is the thinking human being who performs risky actions and decisions whose risk perception influences those actions. Risk perception is thus not only based on personality but also modified by experience, which is crystallized in the form of the implicit representation of risky situations, sources and possible outcomes. Concerning professionally significant risks, professional experience might play a crucial role in the shaping of those representations. Thus, it is the professional (in our case, a doctor) who is the carrier of those representations, arguably presented in the form of “tacit knowledge” and thus difficult to realize and verbalize [22]. 

Thus, to access that knowledge, or specifically representations of medical risk, we used the interviewing procedure and qualitative analysis of the protocols, resulting in a list of 21 risks that reflect the most frequent categories from the practitioners. Those risks are considered important for the evaluation of risk perception among doctors, at least in Russian-speaking samples. The seven quantitative measurements of CRRs aspects were strongly interconnected in our sample of medical practitioners. In our opinion, this indicates the internal structure of cognitive representations of risk, which also bears similarities with experts’ risk-perception, as seen in previous research [32].

We theorize that the probability of a negative outcome, which is correlated with all other CRRs aspects and vigilance, might be one of the core aspects of risk evaluation for medical practitioners. The importance of considering negative outcomes might explain its somewhat contradictory positive correlation with predictability (arguably, the more predictable risk should be easier to prevent, but the results oppose that “common-sense” assumption). Pending further research, we assume that with other professionals or laypeople, this CRRs structure will be different. It is also worth noting that the probability of positive outcome bears little correlation with other CRRs aspects and thus might be related to some other risk evaluation strategies or to being more personality-driven.

The findings from our study extend the data available in the literature, highlighting both women’s sensitivity to risk and the idea that risk probability assessment could be professional experience-related (the decrease was especially consistent for the mean probability of risk in the respondent’s practice) [32].

Moderate correlations of several personality traits with cognitive risk assessments by doctors can be considered in favor of the hypothesis of the unity of functioning of a person’s intellectual and personal potential. However, further research is required in the aspect of the correlation of subjectively perceived and objective risk factors in situations of the professional activity of doctors.

The current study has many limitations, with the sample size being one of the most important. However, the results seem to be meaningful and consistent. For further research, we plan to introduce members of different risky professions into the study as comparison groups.

## Figures and Tables

**Table 1:** Medical risks after categorization (N is the number of units).

Main Category	Subcategories	2nd Level Subcategories	N
1. Doctor-related risks (risks for the doctor and related directly to the doctor’s state or activity) (N = 56)	a. Cognitive risks (medical errors)	i. wrong diagnosis	11
		ii. incorrect assessment of the situation	7
		iii. execution errors	3
	b. Reputational and self-esteem risks	i. reputation losses	7
		ii. self-esteem loss	1
	c. Doctor health risks	i. infection	10
		ii. becoming a victim of aggression	4
		iii. injuries at work	1
	d. Risks of losing time	i. loss of working time	2
		ii. loss of personal time	2
	e. Administrative and legal risks	i. being prosecuted	5
		ii. being subjected to internal sanctions	3
2. Patient-related risks (risks for the patient and related to the patient’s state or activity) (N = 50)	a. Risks of complications		29
	b. Risks of negative effects of treatment		11
	c. Lethal risks		10
3. Risks related to colleagues and institution (N = 9)	a. Colleagues-related risks		3
	b. Boss-related risks		2
	c. Institution-related risks	i. damage to the institution	2
		ii. damage by institution	2
*4. Medicine as science related risks (N = 5)*	*a. Medicine imperfection*		*3*
	*b. Medical research risks*		*2*
5. External risk (N = 3)	e.g., weather, ongoing hostilities		3
*6. Abstract risks (N = 2)*	*e.g., bad luck, chance*		*2*

**Table 2:** Cognitive representations of risks (CRRs) aspects intercorrelations (Spearman’s rank correlation coefficient (rho), significant only).

CRRs Mean Scores and SDs	(2)	(3)	(4)	(5)	(7)
**(1)** Perceived riskiness *(M = 56.40, SD = 16.39)*	0.509 **	0.310 *		0.634 **	0.362 *
**(2)** Predictability *(M = 48.39, SD = 15.98)*		0.552 **	0.465 **	0.425 **	0.454 **
**(3)** Probability in medical practice *(M = 48.93, SD = 20.17)*			0.723 **	0.355 *	0.321 *
**(4)** Probability in the respondent’s practice *(M = 35.65, SD = 17.50)*				0.291 *	0.313 *
**(5)** Perceived emotion depth *(M = 55.61, SD = 19.09)*					0.328 *
**(6)** Positive outcome probability (*M = 53.18, SD = 18.31)*					−0.375 **
**(7)** Negative outcome probability *(M = 48.66, SD = 17.10)*					

* *p* < 0.05; ** *p* < 0.01.

**Table 3:** CRRs aspects correlations with personality traits (Spearman’s rho, significant only).

Personality Traits with Means and SDs	CRRs Aspects			
	(2) Predictability	(3) Probability in Medical Practice	(6) Positive Outcome Probability	(7) Negative Outcome Probability
**(a)** Risk-Readiness *(M = −0.48, SD = 3.16)*	0.291 *			
**(b)** Rationality *(M = 4.94, SD = 3.06)*			−0.341 *	
**(c)** Vigilance *(M = 16.31, SD = 2.05)*			−0.321 *	0.332 *
**(d)** Agreeableness *(M = 8.74, SD = 1.82)*				−0.288 *
**(e)** Intolerance for Ambiguity *(M = 28.90, SD = 7.02)*		−0.315 *		

* *p* < 0.05; ** *p* < 0.01.

**Table 4:** CRRs aspects mean scores in male and female doctors.

CRRs Mean Scores	Female		Male		Mann–Whitney U-test	p
	M	SD	M	SD		
Perceived riskiness	60.71	14.51	48.68	17.10	192.0	0.015
Probability in medical practice	53.17	17.64	41.34	22.58	205.0	0.029
Probability in the respondent’s practice	40.07	16.84	27.74	16.18	165.5	0.003
Perceived emotion depth	62.62	14.64	42.01	19.76	117.5	0.020
Negative outcome probability	52.24	16.25	41.92	17.07	171.0	0.034

**Table 5:** Young, experienced, and older doctors’ personality and CRRs aspects scores.

Variable	Kruskal–Wallis H-test	p	(1) Young		(2) Exp.		(3) Older	
			M	SD	M	SD	M	SD
Risk-Readiness	6.452	0.04	0.95	3.02	−1.17	3.39	−1.16	2.59
Rationality	9.029	0.011 ^1–3^	**3.95**	**3.17**	4.43	3.30	**6.58**	**1.87**
Extraversion	6.477	0.039 ^2–3^	8.80	2.78	**7.78**	**2.68**	**9.84**	**2.29**
Openness	7.643	0.022 ^1–2^	**11.20**	**1.79**	**9.35**	**2.31**	10.26	2.13
Probability in medical practice	6.111	0.047 ^1–3^	**57.49**	**18.47**	46.92	21.23	**40.61**	**17.71**
Probability in the respondent’s practice	10.914	0.004 ^1–2,1–3^	**45.84**	**17.32**	**32.13**	**18.54**	**27.20**	**8.67**
Perceived emotion depth	7.275	0.026 ^1–3^	**59.54**	**19.46**	59.36	16.12	**45.73**	**19.60**

Bold text and superscript text marks pairwise differences (*p* < 0.05), e.g., ^1–3^ means a difference between groups (1) and (3).

## Appendix A

**Table A1:** List of risks for subjective evaluation (translated into English) with related categories from Table 1.

1. Get a penalty	12. Make a mistake while performing a procedure
2. Assess the situation wrong	
3. Ruin the relationship with the boss	13. Get a subpoena
4. Fall from a great height	14. Do not see significant others enough
5. Be a victim of aggression	15. Lose your professional reputation
6. Lose time	16. Harm the patient
7. Lose self-esteem	17. Make a wrong diagnosis
8. Overestimate yourself	18. Break expensive medical equipment
9. Lose the patient	19. Lose health at work
10. Get psychological overload	20. Quarrel with colleagues
11. Have equipment out of order	21. Get into bad weather

## References

[1] Bhise V., Rajan S.S., Sittig D.F., Morgan R.O., Chaudhary P., Singh H. (2017). Defining and measuring diagnostic uncertainty in medicine: A systematic review. J. Gen. Int. Med..

[2] Strout T.D., Hillen M., Gutheil C., Andersen E., Hutchinson R., Ward H., Kay H., Mills G.J., Han P.K.J. (2018). Tolerance of uncertainty: A systematic review of health and healthcare-related outcomes. Patient Educ. Couns..

[3] Kahneman D. (2013). Thinking, Fast and Slow.

[4] Perneger T.V., Agoritsas T. (2011). Doctors and patients’ susceptibility to framing bias: A randomized trial. J. Gen. Int. Med..

[5] Blumenthal-Barby J.S., Krieger H. (2014). Cognitive Biases and heuristics in medical decision making. Med. Decis. Mak..

[6] Donner-Banzhof N., Seidel J., Sikeler A.M., Bösner S., Vogelmeier M., Westram A., Gaissmaier W., Wegwarth O., Gigerenzer G. (2017). The phenomenology of the diagnostic process: A primary care-based survey. Med. Decis. Mak..

[7] Grove M.W., Zald D.H., Lebow B.S., Snitz B.E., Nelson C. (2000). Clinical versus mechanical prediction: A meta-analysis. Psychol. Assess..

[8] Anthony D., Parboteeah S., Saleh M., Papanikolaou P. (2008). Norton, Waterlow and Braden scores: A review of the literature and a comparison between the scores and clinical judgement. J. Clin. Nurs..

[9] McCulloch P., Kaul A., Wagstaff G.F., Wheatcroft J. (2005). Tolerance of uncertainty, extroversion, neuroticism and attitudes to randomized controlled trials among surgeons and physicians. Br. J. Surg..

[10] Mullola S., Hakulinen C., Presseau J., Ruiz de Porras D.G., Jokela M., Hintsa T., Elovainio M. (2018). Personality traits and career choices among physicians in Finland: Employment sector, clinical patient contact, specialty and change of specialty. Bio Med. Cent. Med. Educ..

[11] Pikkel D., Pikkel Igal Y.S., Sharabi-Nov A., Pikkel J. (2016). Are doctors risk takers?. Risk Manag. Healthc. Policy.

[12] Lievens F., Coetsier P., De Fruyt F., Maeseneer J. (2002). Medical students′ personality characteristics and academic performance: A five-factor model perspective. Med. Educ..

[13] Lawton R., Robinson O., Harrison R., Mason S., Conner M., Wilson B. (2019). Are more experienced clinicians better able to tolerate uncertainty and manage risks? A vignette study of doctors in three NHS emergency departments in England. Br. Med. J. Qual. Saf..

[14] Kamenev I., Kornilova T., Razvalyaeva A. (2018). The Relation between Risk Acceptance, Motivation and Self-Regulation (in Sample of Medical Workers). Vopr. Psikhologii.

[15] Kornilova T.V., Chumakova M.A., Kornilov S.A., Novikova M.A. (2010). Psihologiya Neopredelennosti: Edinstvo Intellektual’no-Lichnostnogo Potenciala Cheloveka.

[16] Kornilova T.V. (2016). Intellektualno-Lichnostnyi Potentsial Cheloveka v Usloviyakh Neopredelennosti i Riska.

[17] Slovic P. (2000). The Perception of Risk.

[18] Gigerenzer G. (2015). Simply Rational: Decision Making in the Real World.

[19] Heller R.F., Saltzstein H.D., Caspe V.B. (1992). Heuristics in Medical and Non-Medical Decision-Making. Q. J. Exp. Psychol. A.

[20] Slovic P., Fischhoff B., Lichtenstein S., Schwing R.C., Albers W.A. (1980). Facts and Fears: Understanding Perceived Risk. Societal Risk Assessment: How Safe is Safe Enough?.

[21] Flanagan J.C. (1954). The critical incident technique. Psychol. Bull..

[22] Sternberg R.J., Forsythe G.B., Hedlund J., Horvath J.A., Wagner R.K., Williams W.M., Snook S.A., Grigorenko E. (2000). Practical Intelligence in Everyday Life.

[23] Saunders B., Sim J., Kingstone T., Baker S., Waterfield J., Bartlam B., Burroughs H., Jinks C. (2018). Saturation in qualitative research: Exploring its conceptualization and operationalization. Qual. Quant.

[24] Kvale S. (1996). Interview Views: An Introduction to Qualitative Research Interviewing.

[25] Corbin J., Strauss A. (1998). Basics of Qualitative Research: Techniques and procedures for developing grounded theory.

[26] Mann L., Burnett P., Radford M., Ford S. (1998). The Melbourne Decision Making Questionnaire: An instrument for measuring patterns for coping with decisional conflict. J. Behav. Decis. Mak..

[27] Kornilova T.V. (2013). Melbourne decision making questionnaire: A Russian adaptation. Psihol. Issled..

[28] Gosling S.D., Rentfrow P.J., Swann W.B. (2003). A very brief measure of the Big Five personality domains. J. Res. Pers..

[29] Kornilova T.V., Chumakova M.A. (2016). Development of the Russian version of the brief Big Five questionnaire (TIPI). Psihol. Issled..

[30] Budner S. (1962). Intolerance of ambiguity as a personality variable. J. Pers..

[31] Kornilova T.V., Chumakova M.A. (2014). Tolerance and intolerance of ambiguity in the modification of Budner’s questionnaire. Eksp. Naya Psikhologiya.

[32] Chauvin B., Raue M., Lermer E., Streicher B. (2018). Individual differences in the judgment of risks: Sociodemographic characteristics, cultural orientation, and level of expertise. Psychological Perspectives on Risk and Risk Analysis.

[33] Bogacheva N., Kornilova T., Krasavtseva Y. (2017). Relationships between self-assessments, implicit theories of risk and medical workers’ personal risk readiness. Bull. Marit. Stud. Res. Unit Ser. Psychol..

[34] Donnellan M.B., Lucas R.E. (2008). Age differences in the Big Five across the life span: Evidence from two national samples. Psychol. Aging.

[35] Lauriola M., Weller J., Raue M., Lermer E., Streicher B. (2018). Personality and risk: Beyond daredevils—risk taking from a temperament perspective. Psychological Perspectives on Risk and Risk Analysis.

